# Sub-10-fs observation of bound exciton formation in organic optoelectronic devices

**DOI:** 10.1038/s41467-022-32478-8

**Published:** 2022-08-23

**Authors:** Marios Maimaris, Allan J. Pettipher, Mohammed Azzouzi, Daniel J. Walke, Xijia Zheng, Andrei Gorodetsky, Yifan Dong, Pabitra Shakya Tuladhar, Helder Crespo, Jenny Nelson, John W. G. Tisch, Artem A. Bakulin

**Affiliations:** 1 Department of Chemistry and Centre for Processable Electronics, Imperial College London, London, W12 0BZ UK; 2 Department of Physics, Imperial College London, London, SW7 2AZ UK; 3 Helmholtz Zentrum Berlin für Materialien und Energie, Hahn-Meitner-Platz 1, Berlin, 14109 Germany; 4 School of Physics and Astronomy, University of Birmingham, Birmingham, B15 2TT UK; 5 National Renewable Energy Laboratory, Golden, CO 80401 USA; 6 IFIMUP and Departamento de Física e Astronomia, Faculdade de Ciências, Universidade do Porto, R. do Campo Alegre 687, 4169–007 Porto, Portugal

**Keywords:** Optical spectroscopy, Excited states, Solar cells, Nanoscale devices

## Abstract

Fundamental mechanisms underlying exciton formation in organic semiconductors are complex and elusive as it occurs on ultrashort sub-100-fs timescales. Some fundamental aspects of this process, such as the evolution of exciton binding energy, have not been resolved in time experimentally. Here, we apply a combination of sub-10-fs Pump-Push-Photocurrent, Pump-Push-Photoluminescence, and Pump-Probe spectroscopies to polyfluorene devices to track the ultrafast formation of excitons. While Pump-Probe is sensitive to the total concentration of excited states, Pump-Push-Photocurrent and Pump-Push-Photoluminescence are sensitive to bound states only, providing access to exciton binding dynamics. We find that excitons created by near-absorption-edge photons are intrinsically bound states, or become such within 10 fs after excitation. Meanwhile, excitons with a modest >0.3 eV excess energy can dissociate spontaneously within 50 fs before acquiring bound character. These conclusions are supported by excited-state molecular dynamics simulations and a global kinetic model which quantitatively reproduce experimental data.

## Abstract (web summary)

Ultrafast action spectroscopies of organic optoelectronic devices reveal that the formation of bound exciton state occurs as fast as 10 fs. Excitons having excess energy can dissociate spontaneously within 50-fs before acquiring bound character.

## Introduction

The development of organic molecular-based optoelectronics has been at the forefront of research into green and sustainable energy solutions. Interconversion of photons and molecular electronic states is a key process behind many applications^1,2^, including organic photovoltaics (OPV)^3^, organic light-emitting diodes^4^, light concentrators^5^ and colour convertors^6^. The molecular electronic state formed immediately after photon absorption (and also the precursor state for photon emission) is known as a molecular singlet exciton. Such an exciton is often defined as a quasiparticle consisting of an electron in the lowest unoccupied molecular orbital (LUMO) and a hole in the highest occupied molecular orbital (HOMO). Due to the electrostatic attraction between the localised electron and hole, amplified by the low dielectric constant and low dimensionality^7^, this state is lower in energy compared to a pair of separated charges^3,8^. This makes the electron and hole ‘bound’ to each other, with the energy difference to free carriers known as the exciton binding energy^9^. Exciton binding energy in a solid polymer film depends on a variety of factors, including the dielectric constant^10^, localisation of charges^11,12^, push-pull character^13^, intermolecular coupling, such as electronic coupling^14,15^, molecular aggregation^16^, molecular dimensionality^7^ and morphology^17^. While the high exciton binding energy may be favourable for photo- and electroluminescent applications, the efficient exciton dissociation into separated charges is attractive for photovoltaics. Charge generation in most OPVs is realised using band offsets as the driving force in donor-acceptor materials^18^. However, a ‘spontaneous’ exciton dissociation is also possible and has recently found applications in single component organic solar cells^19,20^, where the molecules of the same type act as both donor and acceptor. Using single-component material reduces the negative effect of exciton diffusion and excess energy loss on the open-circuit voltage of organic photovoltaic devices^21,22^.

The current paradigm for exciton photophysics suggests that immediately after optical excitation an exciton is generated with excess energy (hot exciton) and a delocalised wavefunction across multiple molecules or a single polymer chain^23–26^. This hot exciton then loses its excess energy while localising into a more spatially confined electronic wavefunction. This localisation and relaxation are naturally accompanied by the increase of exciton binding energy, which becomes very substantial in the ‘cold’ state - prohibiting efficient exciton dissociation^12,27–31^. Multiple studies have demonstrated that the excess energy of hot excitons facilitates dissociation into separated charges^12,32^ and shown that higher excitation energy enhances charge separation as excess energy increases the delocalisation character^33–36^. In some materials, however, weakly-bound localised excitons were reported even at room temperatures with dissociation independent of excitation conditions^37–39^. The ambiguous role of the excess excitation energy in exciton binding and dissociation has been also addressed by numerous theoretical studies^40–42^. Despite this effort, due to the complex and exceptionally fast electronic dynamics, the timescales at which excitons form and acquire their properties remain unclear. Overall, the mechanisms and rates of exciton formation and spontaneous dissociation are still widely debated questions that are of immediate importance for the design of optoelectronic materials and devices.

The ultrafast sub-200-fs timescales of bound exciton formation^27,29,43,44^ impose stringent time-resolution requirements on the experimental technique used to study this process which are only attainable by optical techniques. The most common and versatile method used has been ultrafast optical pump-probe (PP) spectroscopy^35,45–47^. Ultrafast PP spectroscopy has proved to be a useful tool to capture exciton dynamics, showing a rapid (within 100-fs time resolution) creation of excitons^45,48^ with an ultrafast (<200 fs) exciton localisation^35^ and charge separation^46,47^. Furthermore, ultrafast transient two-photon photoemission (2PPE) also showed a rapid presence of excitons with localisation occurring in hundreds of femtoseconds through electron-vibrational interaction and nuclear reorganisation^17,49,50^. Previous transient photoluminescence (PL) and transient anisotropy experiments suggest that bound excitons are formed in less than 200 fs^26,27,51,52^ and their binding is predominantly driven by the coupling to phonons and environment^23,25,49,50^. Although these methods track well the population dynamics of states with specific energies, they do not provide direct access to the binding energy of excitonic states and may not distinguish excitons from other excited species. This lack of bound-state sensitivity can be overcome using pump-push-photocurrent (PPPC) and pump-push-photoluminescence (PPPL) techniques^18,53–58^. In PPPC, after triggering exciton formation by initial pump excitation, a push pulse can re-excite bound states promoting their dissociation and the push-induced photocurrent, associated with the *bound states only*, is detected. Similarly, in PPPL, the effect of push on the dissociation of emissive *exciton states only* is detected via measuring the reduction in total luminescence yield of the sample.

Here, we apply a combination of PP, PPPC and PPPL spectroscopies with sub-10-fs time resolution to separate and track in time the ultrafast formation of bound excitons. Using a polyfluorene poly(9,9-dioctylfluorene) (PFO) device as a model system, we use PP to reveal the total concentration of excited states, and PPPC/PPPL to determine the relative number of bound states at different moments of time. We found that excitons created by near-absorption-edge excitation are intrinsically bound states, or are becoming such within 10 fs after excitation. At the same time, hot excitons with >0.3 eV excess energy do have a chance for spontaneous dissociation and acquire fully bound character only at a 50-fs timescale. Excitation fluence-dependent measurements show that the exciton binding does not depend on the density of excited states and that exciton-exciton annihilation contributes to charge dissociation by giving excess energy to the bound exciton. Also, polarisation-sensitive PPPL measurements reveals that energy transfer in PFO occurs on 800-fs clearly slower than exciton binding (<10 fs) and exciton relaxation (∼50 fs). We develop a simple global kinetic model which reproduces PP and PPPC experiments with a compact set of shared parameters, supporting the above conclusions. Finally, we use the non-adiabatic excited state molecular dynamics package (NEXMD)^59^ to simulate singlet exciton evolution in an oligomeric model of this polymer. Our simulation confirms that the binding energy of the near-band-edge S1 excitons does not evolve significantly beyond the ∼20-fs timescale. These near-band-edge states are populated through hot exciton states cooling with characteristic time of 50 fs.

## Results

### PFO material and devices under study

**Fig. 1:**
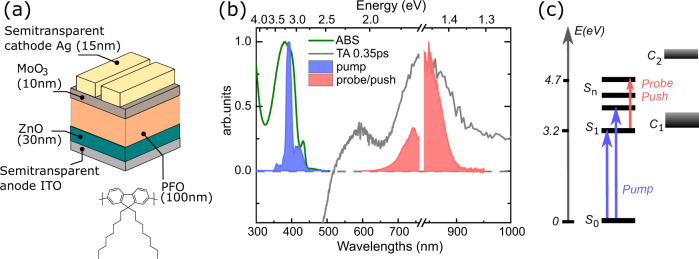
The material system and the device under study. (**a**) Optoelectronic device design and molecular structure of PFO, (**b**) steady state absorption spectrum of PFO (green) and TA spectra (grey) of PFO with 390 nm excitation at 0.35 ps after excitation. The blue area indicates the broad pump pulse and the red area indicates the broad push/probe pulse used in sub-10-fs PP, PPPC and PPPL experiments, (**c**) state energy diagram based on steady state and transient absorption results with So the ground state, S_1_ the singlet state, S_n_ the higher excited states, C_1_ and C_2_ charge separated states (CT or free carriers) and blue and red arrows denoting the pump and push/probe pulse in sub-10-fs PP, PPPC and PPPL experiments.

As a model system in this study, we used poly(9,9-dioctylfluorene) (PFO) shown in Fig. 1a. PFO is a stable emissive conjugated polymer that has been investigated extensively over the last decades using steady-state and conventional time-resolved spectroscopy methods^60–66^. Also, when it is prepared in a relatively amorphous solid state, early (sub-ps) PFO dynamics are not strongly affected by aggregation effects^60^.

To replicate the conditions of an optoelectronic device, PFO was used as the thin (100 nm) active layer of an inverted diode with ITO/ZnO/PFO/MoO_3_/Ag structure as shown in Fig. 1a and in the Supplementary Information (SI). Using a thin active layer allowed us to keep the excitation density homogeneous through the film and minimise charge transport effects in PPPC experiments. The light was coupled into the device from the metal electrode side. Using thin (<20 nm) semi-transparent Ag electrodes, minimises the dispersion experienced by the broadband ultrafast optical pulses and helps to keep the temporal resolution high, although first order of group delay dispersion can be also compensated by chirped mirrors used in the formation of sub-10-fs pulses.

Figure 1b shows the steady state absorption spectrum of the samples. The absorption band is centred at 390 nm and attributed to the π → π^*^ transition, common to most conjugated polymers. Exciting this transition leads to singlet exciton formation; therefore, an excitation (pump) with 390 nm central wavelength was used for all time-resolved experiments. The minor absorption shoulder at 410 nm indicates the presence of a small amount (<6%, based on relative oscillator strength) of PFO beta phase. This beta phase is known to play a role in the energy and charge transfer at ps timescales^66–69^, however, its effect is expected to be negligible on the sub-100-fs dynamics discussed in this work. We also note, our excitation pulse was chosen to match the amorphous phase absorption.

To reveal the energy levels of PFO and identify the key optical transitions relevant for the PPPC and PPPL experiments, we started with a broadband transient absorption (TA) measurement with 200-fs time resolution. Figure 1b shows the TA spectrum at 0.35 ps after 390 nm excitation with a low-fluence pump pulse (<10 μJ/cm^2^ to avoid nonlinear processes). Already at 0.35 ps, pronounced negative and positive areas are observed indicating the rapid formation of excited states. We attribute the negative band below 550 nm to ground state bleaching and stimulated emission of the singlet exciton state of PFO. The wide positive area shows two distinct excited state absorption bands, and global analysis of these data reveals two distinct excited species (Supplementary Figs. 3, 4). The first band around 600 nm was previously assigned to the C_1_ → C_2_ absorption of charge-separated states in the form of either interchain charge-transfer (CT) excitons or polarons (weakly bound or unbound charge carriers)^60,61,67,70,71^. Based on longer relaxation timescales derived from global analysis (Supplementary Fig. 4) and literature^60,61,67^, these charge-separated states are precursors for photocurrent, if they survive recombination. The second band in the far-red and NIR is assigned to the photoexcitation of the singlet exciton to a higher electronic state S_1_ → S_n_^60,61,64,67,70,71^. This assignment agrees with the decay time constant of 200 ps found from global analysis (Supplementary Fig. 4). Figure 1c presents the state energy diagram based on steady state and transient absorption results. This diagram was used to plan sub-10-fs experiments and to develop a kinetic model of excited state dynamics.

### Sub-10-fs time-resolved experiments

To track exciton formation in time, we employed PP, PPPC and PPPL experiments with sub-10-fs pump and push/probe pulses providing ∼10-fs time resolution. The use of such short pulses imposes relatively broadband bandwidths for the excitation and probe/push spectra. Shaded curves in Fig. 1b show the spectra of optical pulses used; PP, PPPC and PPPL experiments have been done at identical conditions with the same pump pulse, while the NIR pulse played the role of either probe or push depending on the type of experiment. The pump pulse had bandwidth of ∼0.4 eV and was centred at 390 nm to achieve excitation of the system into a singlet exciton state while the probe/push pulse was centred at 800 nm with a bandwidth of ∼0.35 eV, matching the excited-state absorption of excitons S_1_ → S_n_. The choice of this probe/push pulse spectrum ensures an interaction only with excitons and eliminates artefacts from other excited species such as polarons and CT states. Polarisation of pump and push were orthogonal, unless stated otherwise. Pulse characterisation and setup outline details can be found in the SI (Supplementary Figs. 1, 2).

**Fig. 2:**
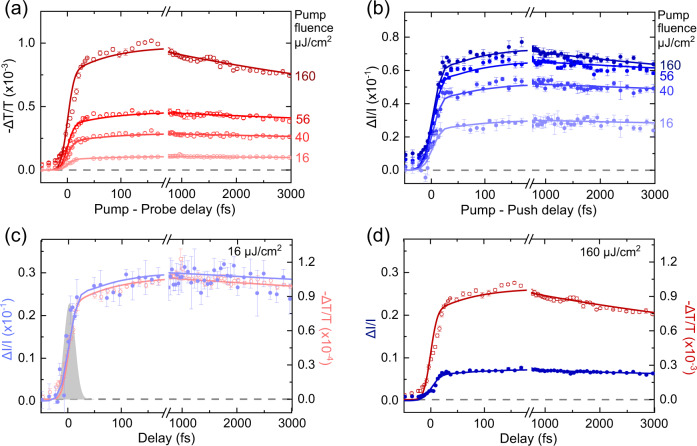
The results of PP and PPPC experiments. (**a**) PP transient transmission (red) and (**b**) PPPC (blue) transient current, for different excitation fluences varying from 16 μJ/cm^2^ up to 160 μJ/cm^2^. Selected PPPC and PP for (**c**) 16 μJ/cm^2^ and (**d**) 160 μJ/cm^2^ plotted on the same graph for comparison. The scales of PPPC and PP for (**d**) 160 μJ/cm^2^ is 10-fold the scales for (**c**) 16 μJ/cm^2^ for better comparison as excitation fluence increases 10-times. Push/probe pulse excitation fluence is kept constant herein. Lines are simulations of the two experiments using the 4-state model illustrated on Fig. 4a. Grey shaded Gaussian on (**c**) represents the convolution of pump and push/probe pulse on PP & PPPC experiments showing the high time resolution here. Error bars are experimental statistical standard errors.

**Fig. 3:**
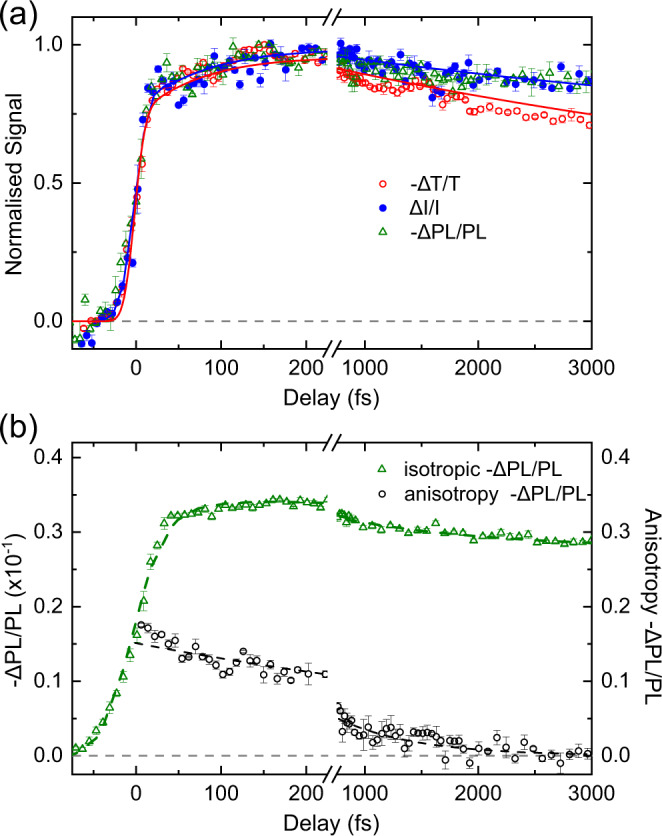
Comparison of the PPPC, PP and polarisation sensitive PPPL experiments. **a** PP transient absorption (red), PPPC (blue) and PPPL (green) at 160 μJ/cm^2^ excitation fluence. Lines are simulations using the 4-state model illustrated on Fig. 4a. **b** Isotropic (green) and anisotropy (black) kinetics of PPPL transient photoluminescence. Dashed lines are exponential fits convoluted with the Gaussian response function. Error bars are experimental statistical standard errors.

**Fig. 4:**
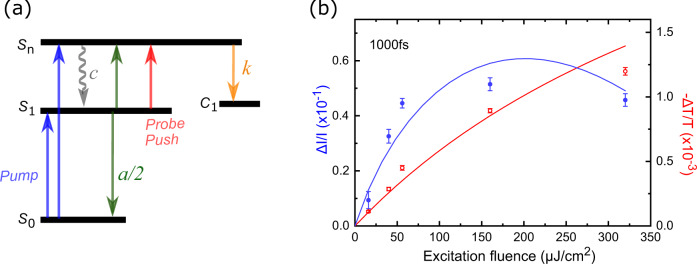
Kinetic modelling of PP and PPPC experiments. **a** kinetic four-state model of PFO ultrafast processes. S0: ground state, S1: singlet exciton state Sn: state representing all higher-energy exciton states, C1: charge separated state precursor of photocurrent, c: cooling, k: charge transfer, a: EEA. The blue and red arrows indicate the pump and push/probe pulses respectively. **b** PPPC (blue) and PP (red) at 1000 fs as a function of excitation fluence; lines are simulations derived from the kinetic four-state model. Error bars are experimental statistical standard errors.

Figure 2 shows PP and PPPC signals for the excitation fluence varying from 16 μJ/cm^2^ up to 160 μJ/cm^2^. Complementary PPPL measurements are shown on Fig. 3 and will be discussed later. The red traces in Fig. 2a show the PP data. PP signal rises abruptly after time zero with 80% of the growth happening within our 10-fs time resolution. This is followed by a slower growth occurring on ∼50-fs timescale. Then, for low excitation fluences, the signal levels off while for higher fluence a noticeable ∼ps decay is observed. As excitation fluence increases, the early PP signal increases roughly linearly with excitation power, but this behaviour does not hold for the long-time signal (Supplementary Fig. 5). Figure 4b shows the PP at 1000 fs for different excitation fluences. The linearity observed up to 100 μJ/cm^2^ has been reported before^61,63^. For stronger intensities, linearity is lost because of the bimolecular effects.

The blue traces in Fig. 2b show the PPPC data. Similar to PP, the transient photocurrent rises very rapidly after time zero with 80% of the growth happening in the first 10-fs followed by slower growth occurring on ∼50 fs timescale. For low excitation fluences, the PPPC signals behave like PP with no pronounced ps decay at longer times (Fig. 2c). However, in contrast to the PP signal, as the excitation fluence intensity increases, the transient photocurrent amplitude achieves saturation (Fig. 4b, Supplementary Fig. 5), and the kinetics shows negligible ps decay even at fluxes above 100 μJ/cm^2^ (Fig. 2b).

The two ultrafast components of PPPC growth suggest that bound exciton formation occurs through two pathways, either a rapid most probable (80%) sub-10-fs pathway, or a slower ∼50-fs process. Since the 50-fs timescale approximately matches previously observed dynamics of spectra diffusion and exciton ‘cooling’ in organic semiconductors^12,21,29,48,65^, we tentatively assign ∼50-fs process to cooling of hot excitons created with higher energy photons. The 10-fs process is then attributed to generation of the cold states with lower energy photons. The existence of both components is expected because a broad pump spectrum is required to achieve a short pulse duration. While most pump photons have their energies within 0.2 eV of the optical gap of PFO, some can populate states with >0.3 eV of excess electronic and vibrational energy.

We note that the interaction of such ultrashort and intense pulses can introduce coherent artefacts and other nonlinear effects. We rule out the influence of these phenomena on our data for two reasons. First, our data have neither an ultrashort spike nor a fast drop of signal within the pulses’ overlap time, which are characteristic for coherent artefact effects. Second, the (sub)linear scaling of PP and PPPC signals with pump intensity within the first 50 fs (Supplementary Fig. 5) rules out multiphoton interaction phenomena.

We associate the ultrafast (sub-10-fs) bound state formation with singlet excitons photogenerated by bringing electrons just above the optical gap. These excitons do not possess significant extra energy above the S_1_ state. As proposed in earlier studies, within the first 10 fs these excitons, which are initially delocalised, begin to localise via vibrational modes that result from the strong electron-phonon coupling. The modes responsible for localisation on such fast timescales are likely to be those responsible for bond length alternation such as the C = C bond stretch and quinoid motion of benzene rings^72^. The period of C = C bond stretch vibration is ∼20 fs which corresponds to the 5-10 fs timescale for a one-way deformation. Indeed, a previous theoretical work found that bond length alternation, which is characteristic of exciton self-trapping, can occur on a 20-fs timescale in polyfluorenes^73^. The sub-10fs timescale does not leave time for slower nuclear reorganisation (via exciton-phonon coupling involving slower motions such as torsion, or via coupling to the environment). Interestingly, the slower nuclear reorganisation processes which follow the initial exciton self-trapping and can take up to 100 ps^74^ appear to have a relatively small impact on the exciton binding energy and the exciton localisation.

We associate the delayed 50-fs growth of signal with bound excitons that form via cooling of directly excited, higher lying states, which may occur via two routes: (i) multiphoton excitation and (ii) high energy photons within the wide spectrum of a short pump pulse. The multiphoton excitation can be discarded as the PP amplitude shows an approximately linear growth at short delay times (Fig. 4b, Supplementary Fig. 5). The broad spectrum of the excitation pulse also allows excitons with few-hundred meV excess energy to be generated. As we show below, additional electronic excited states lie within the pulse range. Some of them may dissociate spontaneously, but some go through the cooling on a 50-fs time scale and then require extra energy for dissociation. The possibility of spontaneous dissociation in the hot state agrees with previously reported 0.3 eV exciton binding energy in PFO^75^. Clark et al have suggested 50-fs time corresponds to S_n_ to S_1_ relaxation in this material^64^.

As pointed out earlier, PP and PPPC kinetics are very similar at low excitation intensities (Fig. 2c), but become different as the excitation intensity increases (Fig. 2d). The differences include the presence of the pronounced ∼ps decay in PP signals and sub-linear behaviour of PPPC amplitude with pump intensity (Fig. 4b). We suggest these two phenomena are connected and originate from exciton-exciton annihilation (EEA) which affects the system significantly at excitation intensities above 100 μJ/cm^2^. In case of PP, the effect is straightforward – at higher pump intensities and exciton densities, EEA opens a new fast decay process observed as a ∼ps decay in the signal^61,63,76^. In case of PPPC, we suggest that EEA gives excess energy to bound excitons and boosts them to overcome the exciton binding energy, offering them the chance to contribute to photocurrent. Therefore, PPPC is not sensitive to the bound excitons undergoing exciton-exciton annihilation as they do not need the push pulse to gain excess energy, and their photocurrent contribution is the same whether there is a push pulse or not. However, EEA depopulates bound states causing the subtle ∼ps decay on high pump intensities PPPC signals.

### Polarisation sensitive measurements

To confirm the attribution of signals to excitons and to evaluate the possible intermolecular energy transfer effect on exciton binding and cooling dynamics in PFO, we also performed polarisation-sensitive PPPL measurements. PPPL were performed on PFO films rather than devices to avoid depolarisation effects in electrode materials and to improve PL outcoupling. The measurements were performed with orthogonal polarisation of pump and push (identical setup to PP and PPPC) as well as with parallel polarisation by adding a 45^o^ polariser in the combined pump/push beam path. Time resolution in parallel polarisation experiment was slightly lower (∼40 fs) due to the dispersion in the polariser.

Figure 3a (Supplementary Fig. 6) shows that, at the orthogonal polarisations, PPPL signal (−ΔPL/PL) follows the same dynamics as PPPC, yet with opposite sign. The matching dynamics verify that, with the chosen push wavelength, PPPC indeed tracks purely singlet excitons, as PPPL addresses only emissive states. Push pulse is absorbed by excitons and splits them into charges, decreasing the PL and increasing the photocurrent of the device; this leads to the opposite signs, but similar dynamics of PPPC and PPPL.

Figure 3b presents the isotropic signal and anisotropy decay observed in PPPL measurements. Signal is strongly depolarised from 10-fs delay times which is likely due to the respective orientation of S_0_ → S_1_ and S_1_ → S_n_ transition dipoles. Strong depolarisation confirms that the orthogonal-polarisation data presented in Fig. 2 are not much affected by polarisation effects. After the initial depolarisation, anisotropy decays further on the timescale of 850 ± 130 fs. We associate these dynamics with the energy transfer process, namely, exciton hoping between the conjugated segments with different orientation. This 800-fs loss of anisotropy is slow compared to the 10-fs bound-exciton formation and to the 50-fs exciton relaxation discussed before. It however matches well the exciton-exciton annihilation dynamics observed in PP measurements. Overall, based on different timescales observed, exciton hoping may only play minor role in the excited state relaxation.

### Kinetic modelling

The above conclusions can be supported by describing the full dataset with a simple kinetic 4-state model illustrated in Fig. 4a. The model comprises the S_0_ ground state, S_1_ singlet exciton state at the optical gap level, S_n_ state representing all higher-energy exciton states, and the charge-separated state C_1_ which is precursor of photocurrent. Following PP and PPPC experiments, after excitation, the system is excited to (mostly) the singlet exciton S_1_ and (less likely) to the higher state S_n_. S_n_ either cools down to the singlet state or dissociates to charges C_1_. We consider EEA and exciton decay (slow but 100% efficient) as the only processes that S_1_ excitons undergo as internal conversion occurs on the >200 ps timescales beyond our experimental window (Supplementary Fig. 4). EEA converts a bound near-band-edge exciton to a hot exciton while another one relaxes to the ground state. Finally, we consider that push pulses interact only with the singlet exciton states, promoting them to the higher electronic exciton state S_n_.

Although the push/probe pulse, EEA, and the high energy part of pump pulse probably promote the system to different higher-energy exciton states, we represent them all by a single higher-energy state S_n_ (Fig. 4a). Such a simplification worked well, helping us to reduce the number of states and free parameters in the differential equations. We assume that S_n_ represents all exciton states lying 0.3 eV above the bandgap. This strong assumption works surprisingly well, indicating that the near-bandgap states are clearly more bound than any higher-lying state.

The rate equations describing this model (1)-(3) include: n_g_ the ground state population, n_n_ the population of higher electronic state, n_1_ the population of singlet exciton state, and n_c_ the population of charge separated states. The rate constants for cooling, charge transfer and EEA are denoted as *c*, *k* and *a*, respectively. These are the key model parameters determined during the global fitting of the data. Exciton generation is described by 10-fs gaussian pulses representing the convolution of sub-10-fs pump and push/probe pulses. Detailed explanation of modelling, fitting and how Δ*Ι/Ι* and −Δ*Τ/Τ* are extracted can be found in the SI.

1 $$\frac{d{n}_{n}}{{dt}}={I}_{{pump}}(t){{\cdot }}\,{n}_{g}(t)-c{{\cdot }}\,{n}_{n}(t)-k{{\cdot }}\,{n}_{n}(t)+a/2{{\cdot }}{n}_{1}^{2}(t)+{I}_{{push}}\left(t\right){{\cdot }}{n}_{1}\left(t\right)$$

2 $$\frac{d{n}_{1}}{{dt}}=-{I}_{{push}}(t){{\cdot }}{n}_{1}(t)+c{{\cdot }}{n}_{n}(t)-a{{\cdot }}{n}_{1}^{2}(t)$$

3 $$\frac{d{n}_{c}}{{dt}}=k{{\cdot }}\,{n}_{n}\left(t\right)$$

The simulation traces are shown in Figs. 2 and 4b as solid lines. The model is able to reproduce the time-dependence and amplitude of the experimental data (5 PP and 5 PPPC kinetics in Fig. 2 and Supplementary Fig. 7), including the saturation behaviour PPPC in Fig. 4b. Therefore, the photophysical picture based on intrinsically bound singlet excitons, enhanced dissociation probability of higher exciton states and EEA, is fully consistent with the reported experimental data.

Fitting showed that *c* is smaller but comparable to *k* (Table 1). Therefore, not only are most excitons bound immediately after excitation (<sub-10 fs), but also the ones that have excess energy and are unbound have limited time for dissociation, meaning, it is hard to ‘harvest’ these excitons while they are hot. The EEA rate coefficient extracted is comparable with the literature^61,63^ (interestingly such EEA rate gives energy transfer times >1 ps, comparable with the timescales observed in PPPL anisotropy measurements). Values of the parameters extracted from the model are shown in Table 1.

**Table 1:** Parameters for cooling, charge transfer, and exciton-exciton annihilation rate for PFO device extracted from fitting and optimisation of the 4-state model to the experimental data

PFO-device	Hot carrier cooling rate (c) (fs^−1^)	Spontaneous hot carrier dissociation rate (k) (fs^−1^)	Exciton-exciton annihilation rate (α) (cm^3^ fs^−1^)
value	0.005	0.01	4 × 10^−24^

### Excited state molecular dynamics modelling

To rationalise the presented results within the frame of current theoretical models, we simulated molecular dynamics and the dynamics of electronic states using the nonadiabatic excited state molecular dynamics (NEXMD) package^59^, which uses a surface-hopping approach to model the evolution of excited states. NEXMD package includes a decoherence correction that overcomes the limitation of surface hopping methods to reproduce coherent vibronic dynamics^59,77,78^. The modelling approach was inspired by the earlier work of Clark et al.^64^ and a pentamer was used to approximate PFO polymer behaviour (see SI for details of non-adiabatic molecular dynamics modelling).

**Fig. 5:**
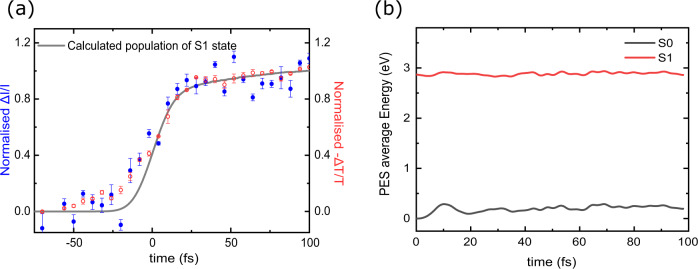
Molecular dynamics modelling of early electronic dynamics in PFO. **a** Molecular dynamics kinetics of first excited state S1 convoluted with the experimental instrument response function (grey line) along with normalised experimental data of PP (red) and PPPC (blue) for low excitation fluences, **b** Average potential energy of S_1_ (red) and S_0_ (black) evolution on the first 100 fs after excitation.

The calculations of electronic excited state energies and absorption strengths indicate that our broadband pump can populate PFO to the first, second and third excited states (Supplementary Fig. 8). Simulations predict that after being instantly excited, higher excited states depopulate within the first 100 fs to the first excited state, S_1_, increasing its population (Supplementary Fig. 9). The total population of state S_1_ convoluted with the instrumental response of the setup is shown in Fig. 5a together with experimental data for pump-push and pump-probe experiments. Good correlation between the modelled and observed kinetics indicates the accurate description of the experiment by theory and confirms that indeed S_1_ is the intrinsically localised state addressed by the experimental techniques we use.

Interestingly, calculations predict that the energy of the S_1_ state does not change substantially in time despite the structural dynamics of polymer chain as Fig. 5b shows. Yet, a significant change of the energy difference of S_1_ and S_n_ (related to exciton binding energy) can be seen only in the first 10 fs. This is in agreement with the experimental observation that the photogenerated exciton becomes bound within the first 10 fs after excitation.

## Discussion

While the existing literature does not contain many experimental reports about the dynamics of excited states’ binding energies, our outcome agrees, for example, with previous 2PPE measurements where no substantial shift in the energy of the excited states was observed within ∼100-fs time resolution^49^. On the contrary, some previous time-resolved PL anisotropy experiments (done on more complex material systems) have reported ∼100 fs and ∼1 ps components in exciton dynamics, assigning the latter to bound exciton formation^28^. Our results point towards possible reinterpretation of these data, suggesting the fast component responsible for exciton binding and the slower dynamics to hot-exciton cooling. The assignment of the fast component to exciton self-trapping via activation of modes that affect local bond length alternation aligns well with theoretical studies that revealed ∼10-fs fluorescence depolarisation due to the exciton-polaron formation assisted by C-C bond vibronic coupling^25,79^.

Our work sheds new light on the ultrafast exciton dynamics and proposes a new methodology to study excitons in the organic systems in the future. The developed approach provides a unique insight into the evolution of exciton binding energy on the 10-fs timescales inaccessible by other methods, like 2PPE. Organic semiconductors are different and addressing their ultrafast processes can give more insight into the parameters which define the exciton fate. For instance, herein, we see that, surprisingly, nuclear reorganisation does not affect bound exciton formation in PFO. We also observe that hot exciton cooling is extremely fast in this polymer – faster than spontaneous dissociation and much faster than exciton hopping. Such fast exciton cooling would be likely to influence the efficiency of charge generation in photovoltaic or photodetector devices. While the picture is specific for PFO and the results cannot be directly generalised for other organic semiconductors, our study answers a long-standing question about how fast exciton is formed and how much structural dynamics contributes to exciton binding. It also highlights the strength of the sub-10-fs PPPC/PPPL techniques in combination with the PP technique for tracking excited states’ evolution on the potential energy surfaces. This can be applied further in low-offset heterojunction and single-component photovoltaic devices to address outstanding questions such as the importance of hot-carrier pathways in charge generation.

In conclusion, we studied the ultrafast exciton dynamics of PFO using a new optical-control approach. Combining sub-10-fs PP spectroscopy, sensitive to all excited states, and sub-10-fs PPPC and PPPL spectroscopies, sensitive to bound states, we separate and track in time the ultrafast formation of bound excitons. Our results imply that a near-band-edge exciton is bound from its very beginning, or it becomes bound on an extremely fast timescale of <10-fs. Hot excitons with excess energy >0.3 eV have the possibility to dissociate into separated carriers or cool down to a near-bandgap state on a 50-fs timescale. Regardless of excitation fluence, exciton binding does not depend on the density of excited states, yet the exciton-exciton annihilation contributes to charge dissociation by re-creating hot excited states. Also, energy transfer in PFO occurs on 800-fs clearly slower than exciton binding (<10 fs) and exciton relaxation (∼50 fs). Finally, to enhance the analysis and findings, a global kinetic model was developed and with a compact set of shared parameters reproduces PP and PPPC experiments and verifies above conclusions. The presented approach and findings are of relevance to the ongoing development of organic materials with low driving energy for charge separation, where quasi-spontaneous dissociation of exciton states is essential for efficient performance of optoelectronic devices.

## Methods

### Device fabrication

The sample is an inverted device with ITO/ ZnO/ F8 (annealed)/MoO3/Ag transparent device structure. The devices were fabricated on ITO coated transparent glass substrate with sheet resistance 15 ohm/square. The ITO substrates were sequentially cleaned by sonication with detergent, deionized water, acetone, and isopropanol, followed by oxygen plasma treatment. Onto the plasma treated ITO substrate the ZnO precursor solution is deposited by spin-coating a zinc acetate dihydrate precursor solution (60.4 μL 1-ethanolamine in 2 mL 2-methoxyethanol) followed by annealing at 150 °C for 10 min, yielding 30 nm of a ZnO. The active layer solution of F8 is made in Chlorobenzene with concentration of 40 mg/ml in the glovebox. The active layer of F8 is spin coated on to the ZnO layer and annealed at 145 C to achieve 100 nm in the inert atmosphere. Following the deposition of the active layers, MoO3 (10 nm) and Ag (15 nm) layers were deposited by evaporation through a shadow mask yielding active areas of 0.045 cm^2^ in each pixel yielding a transparent device.

### Spectroscopy characterization

#### Linear optical characterization

UV-Vis absorption spectra of the PFO sample were obtained using a Shimadzu 2600 spectrometer equipped with an ISR-2600Plus integrating sphere attachment. The slit width was 5 nm and the sampling interval was 1 nm.

#### Transient absorption with 200-fs time resolution

A commercially available broadband pump-probe femtosecond (fs) transient absorption spectrometer Helios (Spectra Physics, Newport Corp.) was used. Ultrafast laser pulses (800 nm, 100 fs duration) were generated by a 1 kHz Ti:sapphire regenerative amplifier (Solstice, Spectra Physics, Newport Corp.). One portion of the 800 nm pulse was directed to an optical parametric amplifier (TOPAS Prime, Spectra-Physics) and a frequency mixer (Niruvis, Light Conversion) to tune the visible pump pulses to 390 nm. The pump pulses were modulated at a frequency of 500 Hz by a mechanical chopper. The rest of the 800 nm pulse was routed onto a mechanical delay stage with a 6 ns time window and directed through a non-linear crystal (sapphire for the visible region and YAG for the NIR region) to generate a white light probe ranging from 400 to 1600 nm. The probe pulse was split into two by a neutral density filter. One portion of the probe pulse served as the reference and was directly sent to the fiber-optic coupled multichannel spectrometers (CCD and InGaAs sensors). The rest of the probe pulse together with the pump pulse were focused onto the same spot on the samples with a beam size of around 0.5 mm^2^ before sending it to the spectrometer. To compensate the fluctuations, the measured spectrum was normalized to the reference spectrum and averaged for several scans to achieve a good signal-to-noise ratio. Data analysis was performed with the commercialized Surface Xplorer software unless otherwise stated. The pump pulse fluence was <10μJ/cm^2^ to avoid non-linear processes. The PFO sample was kept under nitrogen during measurements.

#### Sub-10-fs PP, PPPC and PPPL

A commercial hollow-fibre pulse compression (HFPC) system (ICON, Imperial College) was used to generate sub-10-fs pulses from a 800 nm, 50 fs Ti:sapphire laser (Coherent Astrella, 2.5 W, 4 kHz pulse repetition rate). The 50 fs pulses, at an average power of 1.25 W (∼300 μJ), were coupled into a 250 µm diameter differentially-pumped hollow-core fibre (evacuated with a vacuum pump at the fibre entrance and filled with argon at 0.9 bar at the fibre exit) to spectrally broaden the pulse. The output pulses from the fibre have photon energies in the range 1.4–1.9 eV (650–900 nm). Temporal compression to sub-10-fs duration was achieved via dispersion compensation from 10 bounces off chirped mirrors (Layertec, 40 fs^2^ per bounce). The compressed pulses were split approximately 50/50 into two arms using a d-cut mirror. The first arm is the probe/push arm where the sub-10-fs pulse serves as the probe/push pulse and passes through a micrometre-precision delay stage (*LNR50 Series Encoded, Linear, Long-travel Translation Stage, Thorlabs*). The second arm is the pump arm where a 50 µm BBO crystal is used to generate a pump pulse centred around 3.1 eV (400 nm) using Type-I second harmonic generation. Another set of chirped mirrors (Ultrafast Innovations, 50 fs^2^ per bounce) was used to compensate the 400 nm beam for dispersion in the BBO crystal and the air path, resulting in sub-10-fs pump pulses. A waveplate was inserted before the d-cut mirror to ensure that the polarisation of the second harmonic was orientated correctly for the pump-chirped mirrors. The dispersion in each of the arms is independently optimised by fine tuning the amount of glass in the optical path via two sets of AR-coated glass wedges mounted on translation stages. The two beams (pump and probe/push) were then recombined using a second d-cut mirror and focused into the sample device with a f = 30 cm focusing mirror using a small angle of incidence (<5°) to minimise astigmatism. Photodetectors detected the transmitted probe pulse for PP experiments and the sample’s photoluminescence for PPPL experiments, while for PPPC experiments, the sample device was connected to a photocurrent detector (MFLI Lock-in Amplifier, Zurich Instruments Ltd.). A chopper wheel was placed either in the probe/push arm or the pump arm for PPPL/PPPC and PP experiments, respectively.

### Molecular dynamics

Molecular dynamics were performed using the nonadiabatic excited state molecular dynamics (NEXMD) package. For the simulation of the absorption spectrum of the polymer, we first get the optimised geometry of the pentamer using the Austin Model 1 (AM1) semiempirical model Hamiltonian. Then we calculate a ground state trajectory, to generate the different initial geometries of the molecule. We do an AM1 ground state molecular dynamics simulation of 1 ps long at 300 K with time steps of 0.1 fs. The Langevin thermostat was used to keep the temperature constant with a friction coefficient ζ = 2.0 ps-1. These trajectories were used to collect as set of initial positions for the subsequent excited state calculation. We used 1000 different initial positions and the subsequent data shown are an average of all these trajectories.

## Supplementary information

Supplementary Information

## Data Availability

The datasets generated during and/or analysed during the current study are available from the corresponding author on reasonable request.
