# Supplementary material for: Sub-10-fs observation of bound exciton formation in organic optoelectronic devices
Source: Nat Commun. 2022 Aug 23;13:4949. doi: 10.1038/s41467-022-32478-8 (PMC9399228; doi:10.1038/s41467-022-32478-8)
Supplement: Supplementary file 1 — Supplementary Information [file 41467_2022_32478_MOESM1_ESM.pdf]

Supplementary Information for

Sub-10-fs Observation of Bound Exciton Formation in Organic Optoelectronic Devices

Marios Maimaris[1,], Allan J. Pettipher[2], Mohammed Azzouzi[2], Daniel J. Walke[2,3], Xijia Zheng[1], Andrei Gorodetsky [1,4], Yifan Dong[1,5], Pabitra Shakya Tuladhar[1], Helder Crespo[2,6], Jenny Nelson[2], John W. G. Tisch[2], Artem A. Bakulin[1]*

[1] Department of Chemistry, Imperial College London, London W12 0BZ, UK

[2] Department of Physics, Imperial College London, London SW7 2AZ, UK

[3] Helmholtz Zentrum Berlin für Materialien und Energie, Hahn-Meitner-Platz 1, 14109 Berlin, Germany

[4] School of Physics and Astronomy, University of Birmingham, Birmingham B15 2TT, UK

[5] National Renewable Energy Laboratory, Golden, CO 80401, USA

[6] IFIMUP and Departamento de Física e Astronomia, Faculdade de Ciências, Universidade do Porto, R. do Campo Alegre 687, 4169–007 Porto, Portugal

*Corresponding author e-mail: marios.maimaris17@imperial.ac.uk

Experimental Methods

Materials. The sample is an inverted device with ITO/ ZnO/ F8 (annealed)/MoO3/Ag transparent device structure. Fabrication details follows: The devices were fabricated on ITO coated transparent glass substrate with sheet resistance 15 ohm/square. The ITO substrates were sequentially cleaned by sonication with detergent, deionized water, acetone, and isopropanol, followed by oxygen plasma treatment. Onto the plasma treated ITO substrate the ZnO precursor solution is deposited by spin-coating a zinc acetate dihydrate precursor solution (60.4 µL 1-ethanolamine in 2 mL 2-methoxyethanol) followed by annealing at 150 °C for 10 min, yielding 30nm of a ZnO. The active layer solution of F8 is made in Chlorobenzene with concentration of 40 mg/ml in the glovebox. The active layer of F8 is spin coated on to the ZnO layer and annealed at 145C to achieve xx nm in the inert atmosphere. Following the deposition of the active layers, MoO3 (10 nm) and Ag (15 nm) layers were deposited by evaporation through a shadow mask yielding active areas of 0.045 cm2 in each pixel yielding a transparent device.

Linear optical characterization. UV-Vis absorption spectra of the PFO sample were obtained using a Shimadzu 2600 spectrometer equipped with an ISR-2600Plus integrating sphere attachment. The slit width was 5 nm and the sampling interval was 1 nm.

Transient absorption with 200-fs time resolution. A commercially available broadband pump-probe femtosecond (fs) transient absorption spectrometer Helios (Spectra Physics, Newport Corp.) was used to measure the transient absorption spectra and kinetics for PFO thin film samples. Ultrafast laser pulses (800 nm, 100 fs duration) were generated by a 1 kHz Ti:sapphire regenerative amplifier (Solstice, Spectra Physics, Newport Corp.). One portion of the 800 nmpulse was directed to an optical parametric amplifier (TOPAS Prime, Spectra-Physics) and a frequency mixer (Niruvis, Light Conversion) to tune the visible pump pulses to

390 nm. The pump pulses were modulated at a frequency of 500 Hz by a mechanical chopper. The rest of the 800 nm pulse was routed onto a mechanical delay stage with a 6 ns time window and directed through a non-linear crystal (sapphire for the visible region and YAG for the NIR region) to generate a white light probe ranging from 400 – 1600 nm. The probe pulse was split into two by a neutral density filter. One portion of the probe pulse served as the reference and was directly sent to the fiber-optic coupled multichannel spectrometers (CCD and InGaAs sensors). The rest of the probe pulse together with the pump pulse were focused onto the same spot on the samples with a beam size of around 0.5 mm^2 before sending it to the spectrometer. To compensate the fluctuations, the measured spectrum was normalized to the reference spectrum and averaged for several scans to achieve a good signal-to-noise ratio. Data analysis was performed with the commercialized Surface Xplorer software unless otherwise stated. The pump pulse fluence was <10μJ/cm^2 to avoid non-linear processes. The PFO sample was kept under nitrogen during measurements.

Sub-10-fs PP, PPPC and PPPL experimental apparatus.

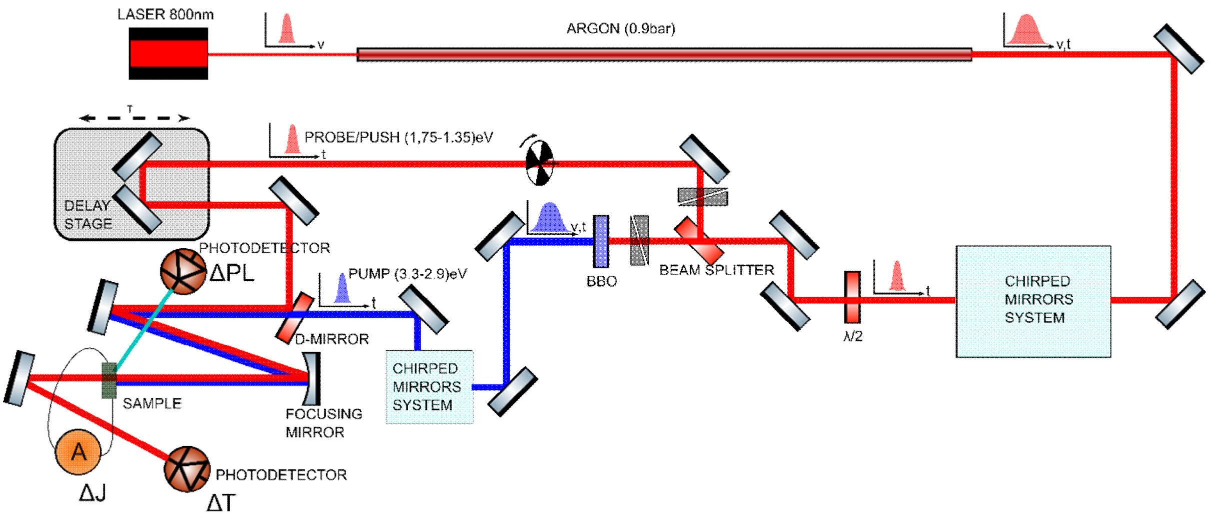

Supplementary Fig. 1*. Experimental apparatus of sub-10fs PP, PPPC and PPPL experiments.*

Supplementary Fig. 1 shows the experimental apparatus for sub-10-fs PP, PPPC and PPPL. A commercial hollow-fibre pulse compression (HFPC) system (ICON, Imperial College) was used to generate sub-10-fs pulses from a 800 nm, 50 fs Ti:sapphire laser (Coherent Astrella, 2.5 W, 4 kHz pulse repetition rate). The 50 fs pulses, at an average power of 1.25 W (~300 μJ), were coupled into a 250 μm diameter differentially-pumped hollow-core fibre (evacuated with a vacuum pump at the fibre entrance and filled with argon at 0.9 bar at the fibre exit) to spectrally broaden the pulse, thus permitting pulse temporal compression. The HFPC system uses beam-pointing stabilisation to lock the position and angle of the input beam to the entrance of the fibre to ensure the long-term stability required for lengthy data scans. The output pulses from the fibre have photon energies in the range 1.4-1.9 eV (650-900 nm). Temporal compression to sub-10-fs duration was achieved via dispersion compensation from 10 bounces off chirped mirrors (Layertec, 40 fs^2 per bounce). The compressed pulses were split approximately 50/50 into two arms using a d-cut mirror. The first arm is the probe/push arm where the sub-10-fs pulse serves as the probe/push pulse and passes through a micrometre-

precision delay stage (*LNR50 Series Encoded, Linear, Long-travel Translation Stage, Thorlabs*) to control the time delay between the pump and probe/push pulses. The second arm is the pump arm where a 50 μm BBO crystal is used to generate a pump pulse centred around 3.1 eV (400 nm) using Type-I second harmonic generation. Another set of chirped mirrors (Ultrafast Innovations, 50 fs^2 per bounce) was used to compensate the 400 nm beam for dispersion in the BBO crystal and the air path, resulting in sub-10-fs pump pulses. A waveplate was inserted before the d-cut mirror to ensure that the polarisation of the second harmonic was orientated correctly for the pump chirped mirrors. The dispersion in each of the arms is independently optimised by fine tuning the amount of glass in the optical path via two sets of AR coated glass wedges mounted on translation stages. The two beams (pump and probe/push) were then recombined using a second d-cut mirror and focused into the sample-device with a f = 30 cm focusing mirror using a small angle of incidence (<5°) to minimise astigmatism. Photodetectors detected the transmitted probe pulse for PP experiments and the sample's photoluminescence for PPPL experiments, while for PPPC experiments, the sample-device was connected to a photocurrent detector (MFLI Lock-in Amplifier, Zurich Instruments Ltd.). A chopper wheel was placed either in the probe/push arm or the pump arm for PPPL/PPPC and PP experiments, respectively. Reflective optical components were used to avoid additional dispersion compensation and to preserve the temporal resolution crucial for tracking bound exciton formation

Supplementary Fig. 2 shows the spectra of pump and probe/push pulses used in this study and generated with the above experimental apparatus.

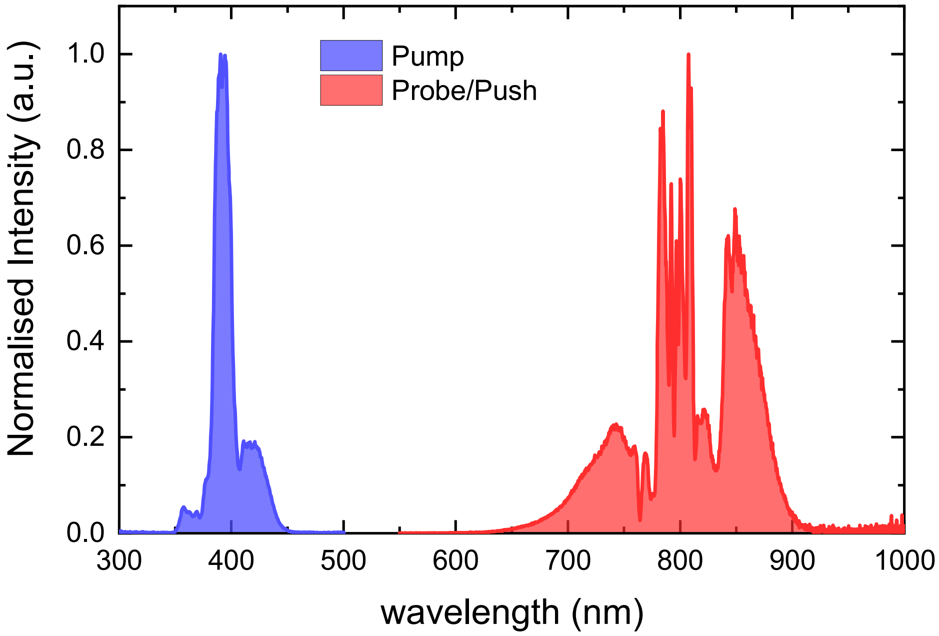

Supplementary Fig. 2. *Normalised pump (blue) and probe/push (red) pulses spectra.*

The push pulse duration was measured to be 8.4 fs FWHM using the d-scan technique[1,2]. A cross-correlation of the pump and probe/push pulses in the sample was carried out, giving a cross-correlation width of below 10 fs; therefore, a sub-10-fs time resolution is achieved in PP, PPPC and PPPL.

Time Resolved Spectroscopy

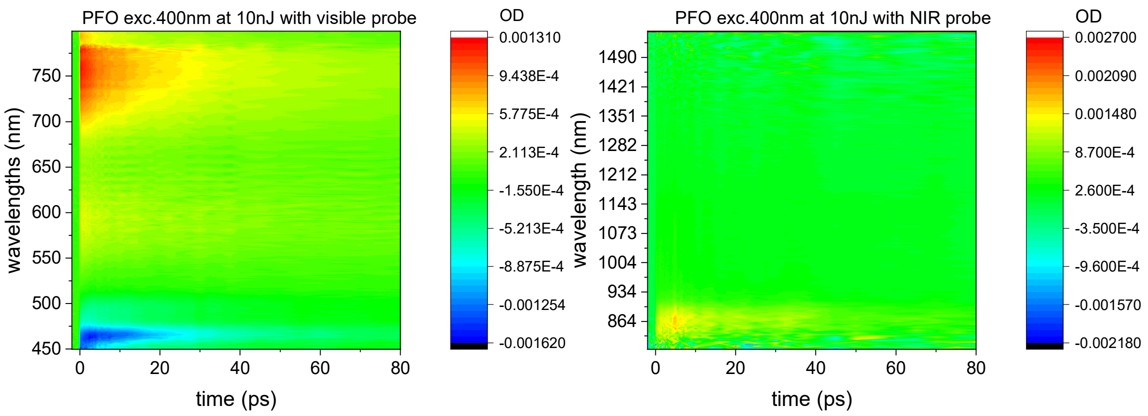

Supplementary Fig. 3. *2D map of Transient Absorption spectroscopy with >200fs time resolution of PFO excited by 400nm and 10nJ pulse energy, left: detection in visible range, right: detection in NIR.*

Supplementary Fig. 3 shows the 2D maps of Transient Absorption (TA) spectroscopy with >200fs time resolution of PFO. In the visible detection two positive and one negative band are observed. In the NIR only one positive band is observed which is the same with the positive band in longer wavelengths with the visible detection. The negative band corresponds to stimulated emission and ground state bleaching.

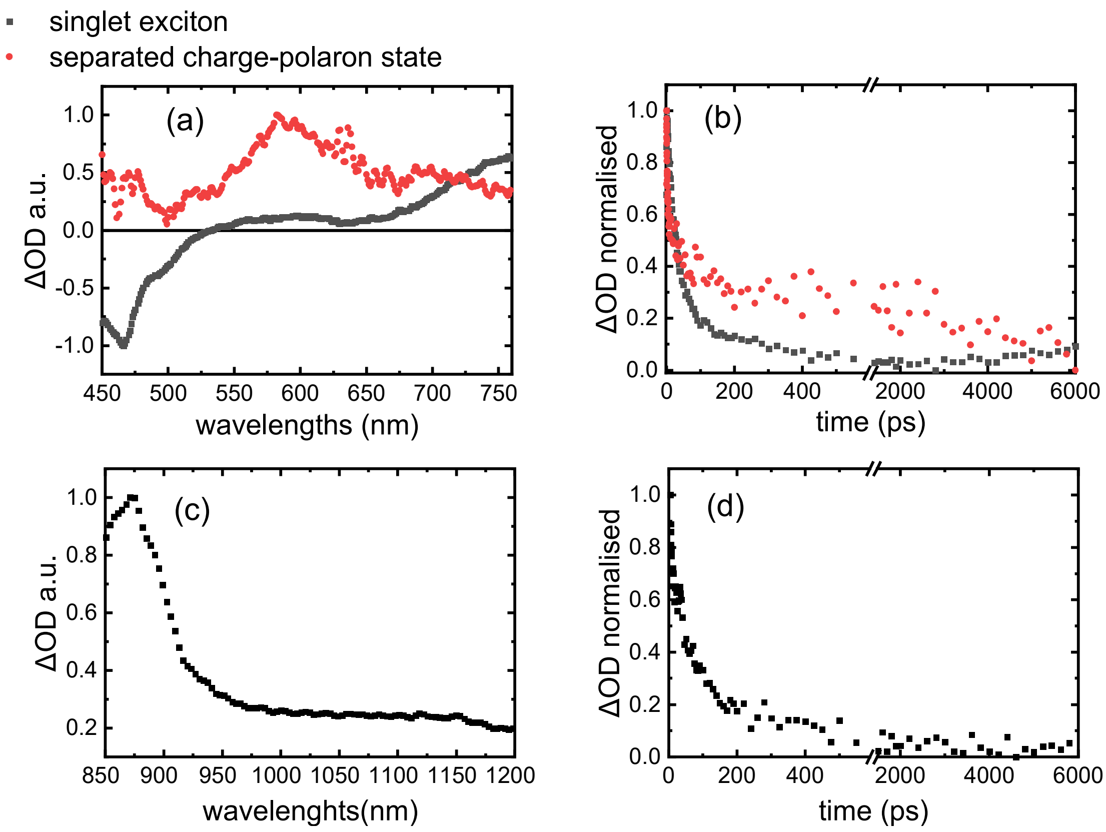

Supplementary Fig. 4. *The 2D map global analysis results. a) two components from global analysis of TA in the visible area. b) dynamics of the two components in the visible range, c) single component from global analysis of TA in the NIR area, d) dynamics of the NIR component.*

Supplementary Fig. 4 shows global analysis of the 2D map of Supplementary Fig. 3. There are two main components. The first is long-lived, causing the positive band around 600 nm and is assigned to charge separated states. The second has a shorter lifetime around 200 ps and causes the negative band of bleaching and stimulated emission; therefore, it is assigned to excitons. A lifetime of 200 ps supports the omission of exciton relaxation in the experimental and theoretical analysis of sub-10 fs PP and PPPC.

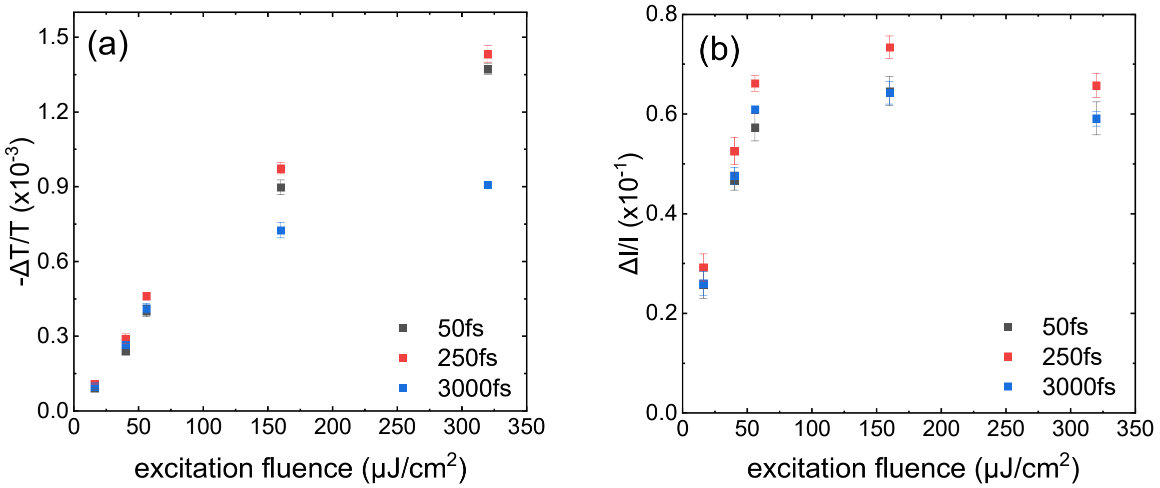

Supplementary Fig. 5. *-ΔT/T (a) and ΔPC/PC (b) at 50fs, 250fs, and 3000fs after pump excitation as a function of excitation flux intensity. Error bars are experimental statistical standard errors.*

In Supplementary Fig. 5 PP and PPPC signals for 50fs, 250fs, and 3000fs for increased excitation fluence is shown. Linear response is observed mainly below 100μJ/cm^2 and agrees with Cheetham et al. 2019. For higher excitation flux intensities there is a sub-linear response for -ΔT/T and a saturation for ΔPC/PC. Evidence of sub-linear response and absence of super-linearity in -ΔT/T erases multiphoton absorption of pump pulse. The already high signal of ΔI/I at 50fs and similar to 250fs supports the idea of ultrafast bound excitons. Also, the increase between 50fs and 250fs observed in both -ΔT/T and ΔPC/PC signals reveals the existence of another mechanism of exciton binding yet this increase is relatively small compared to the signal at 50fs showing that the fast growth is the dominant mechanism of exciton binding.

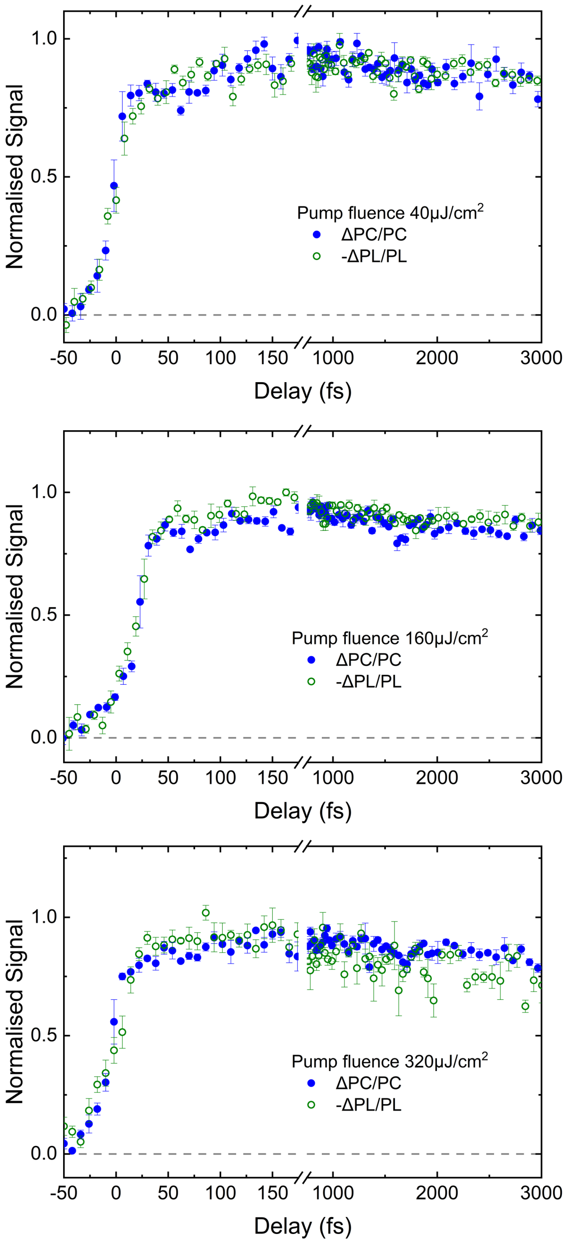

Supplementary Fig. 6. *PPPC (blue) and PPPL (green) transients at different excitation fluences at orthogonal polarisation of pump and push beams.*

Supplementary Fig. 6 shows that PPPC (blue) and PPPL (green) depict similar dynamics at different excitation fluences. This verifies that, the PPPC and the PPPL experiments push and track same excited state, that is, purely singlet excitons, as PPPL addresses only emissive states. The negative sign at PPPL occurs because push pulse is absorbed by excitons and splits them into charges, decreasing the PL.

Modelling

$$\frac{dn_0}{dt} = -I_{pump}(t)\cdot n_g + \; a/2\cdot n_1^2 \tag{1}$$

$$\frac{dn_n}{dt} = I_{pump}(t)\cdot n_g - c\cdot n_n - k\cdot n_n + \; a/2\cdot n_1^2 \; + \; I_{push}(t,\tau_{delay})\cdot n_1 \tag{2}$$

$$\frac{dn_1}{dt} = -I_{push}\;(t,\tau_{delay})\cdot n_1 + c\cdot n_n - a\cdot n_1^2 \tag{3}$$

$$\frac{dn_c}{dt} = k\cdot n_n \tag{4}$$

The equations above describe the model in Figure 3a, where n_g the ground state population, n_n the population of higher electronic state, n_1 the population of singlet exciton state and n_c the population of charge separated states. The rate constants for cooling, charge transfer and exciton-exciton annihilation are denoted as c, k and, a, respectively. For the pump and push term, $I_{pump}(t)$, $I_{push}\;(t,\tau_{delay})$, gaussian functions are used with σ =10fs. The push term is also a function of τ_{delay} which is assigned to the pump-push delay-time. The recombination rate of singlet exciton is omitted because of its long timescale (derived from TA data) compared to the short timescales under investigation.

Inclusion of the non-linear exciton-exciton annihilation term which cannot be neglected in high pump fluences, requires that the coupled differential equations are solved numerically, and so both low and high excitation fluences are numerically solved using midpoint method implemented in python programming.

To simulate the PP experiment the above differential equation system is solved without the term describing push-pulse, $I_{push}\;(t,\tau_{delay})$. The time evolution of n_1 state, that is $n_1(t)$, describes kinetics of the singlet exciton state. In other words, $n_1(t) \propto \Delta T/T$.

To simulate the PPPC experiment, the analysis is not so straight forward, as $I_{push}\ (t, \tau_{delay})$ term needs to be included which contains the τ_{delay}.

We assume that the only states which contributes to photocurrent are the charge separated states n_c. Then two problems emerge. We cannot know (i) the exact percentage of separated charges contributing to photocurrent and (ii) the exact time at which the separated charges are extracted for the photocurrent after their generation. However, since PPPC is the transient photocurrent, (i) is eliminated as we assume that the percentage of separated charges contributing to photocurrent is the same regardless of the charge separated states density. The (ii) can be arbitrary as the same extraction time is taken whether push-pulse is present or not. Herein we use t = 6ps as the extraction time.

To derive from the modelling the PPPC signal as a function of the τ_{delay}, we need to calculate the population of n_c at 6ps with the push-term at each τ_{delay} and without the push-term. The difference of these two populations for each τ_{delay} is proportional to the PPPC signal at this τ_{delay}. In other words, $n_{cwithpush}\left(t = 6ps, \tau_{delay}\right) - n_{cwithoutpush}(t = 6ps) \propto \Delta I / I(\tau_{delay})$

For example, to calculate the PPPC at $\tau_{delay} = 500fs$, we need a) to use $I_{push}(t, \tau_{delay} = 500fs)$ and then solve the above system of differential equation to derive the $n_{cwithpush}(t, \tau_{delay}\ = 500fs)$ and b) to solve the system without the push-term to derive the $n_{cwithoutpush}\ (t)$. Then, the final step is to set $t = 6ps$ and do the subtraction $n_{cwithpush}\left(t = 6ps, \tau_{delay} = 500fs\right) - n_{cwithoutpush}(t = 6ps)$ which is proportional to the $PPPC\ (\tau_{delay} = 500fs)$ signal. To construct the PPPC kinetics, above steps needs to be done for all τ_{delay}.

Then theoretical PP and PPPC data are fitted to the experimental PP and PPPC data to investigate the validity of the model and extract the fitted parameters of *c*, *k* and, *a*.

Global fitting using pairs of PP and PPPC of similar pump fluence gives the fitting traces shown on Figure 2. Here global fitting was undertaken with *c*, *k* and, *a* as shared parameters and push and pump excitation term to be free parameters. For the Figure 3b the global fitting was undertaken with the same conditions but having now the push-term constant. Although pump-term was a free parameter, global fitting showed that the value of the pump-term was increasing proportionally to the experimental pump fluence as we fitted higher pump-fluences.

We note that for higher fluences the model starts to deviate and is not adequate to describe experimental data as other non-linear mechanisms are expected to contribute significantly e.g., exciton-polaron annihilation. Supplementary Fig. 7 shows this deviation at higher excitation fluences.

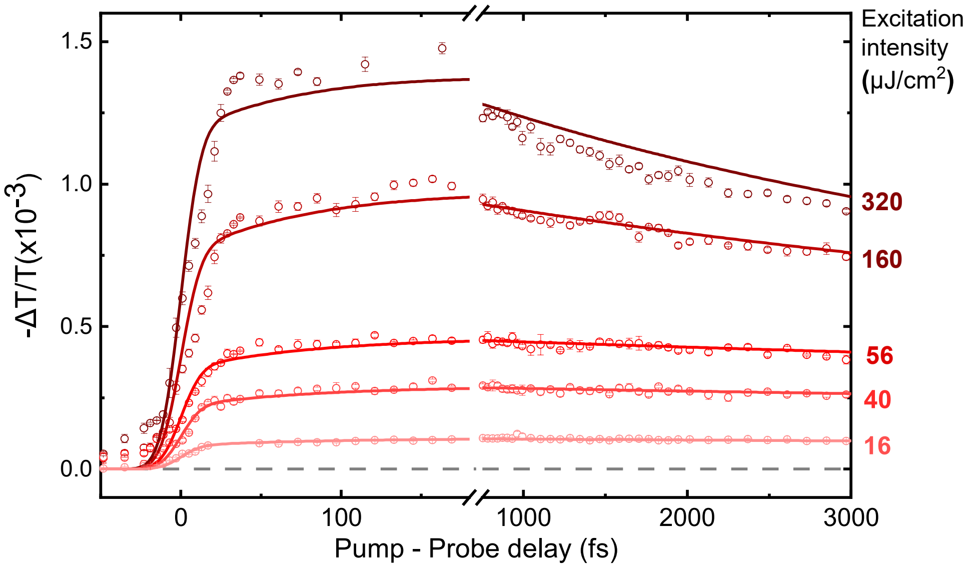

Supplementary Fig. 7. *PP transient transmission for different excitation fluences. Excitation fluence varies from 16 μJ/cm^2 up to 320 μJ/cm^2.*

Molecular Dynamics

For the quantum calculation we use the NEXMD program[3,4]. We use the program to both simulate the absorption spectrum of the PFO polymer as well as its dynamics in the first few 100 ps following a photoexcitation. We use a PFO pentamer for all the calculations below.

For the simulation of the absorption spectrum of the polymer, we first get the optimised geometry of the pentamer using the Austin Model 1 (AM1) semiempirical model Hamiltonian following the method used by Clark et al[5]. Then we calculate a ground state trajectory, to generate the different initial geometries of the molecule. We do an AM1 ground state molecular dynamics simulation of 1 ps long at 300K with time steps of 0.1fs. The Langevin thermostat was used to keep the temperature constant with a friction coefficient $\zeta = 2.0$ ps-1. These trajectories were used to collect as set of initial positions for the subsequent excited state calculation. We used 1000 different initial positions and the subsequent data shown are an average of all these trajectories.

The optical absorption of the PFO pentamer is shown in Supplementary Fig. 8, with the absorption of the different electronic excited states shown in different colours. Here we only show the absorption of the first 4 excited states, as they are the ones absorbing in the region of the pump laser. Considering the level of theory and the simplified system simulated, the agreement with the experiment is not bad. The results in Supplementary Fig. 8 show that the pump laser will mainly excite the S1 state, but we cannot neglect the contribution from the S2 and S3 states.

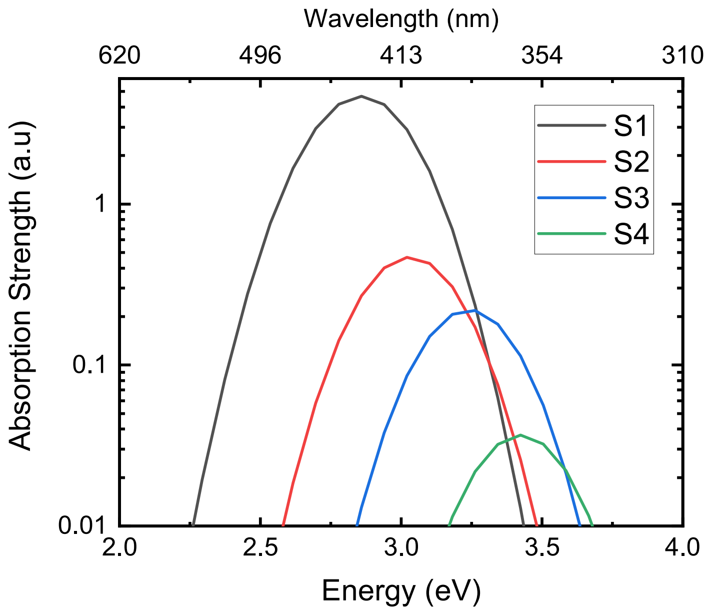

Supplementary Fig. 8. *Calculated ground state absorption of the PFO pentamer. Molecular dynamics show that pump pulse can populate the 4 lowest energy excited states.*[4]

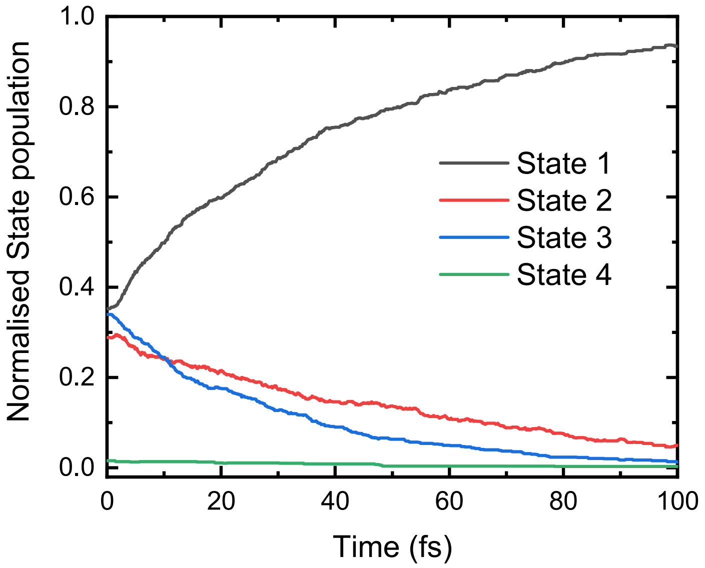

Supplementary Fig. 9. *Simulated Excited state dynamics of a PFO pentamer following an excitation of 3.2eV.*

Supplementary Fig. 9 shows the kinetics of the first 5 excited states. We focus on S1, S2 and S3 excited states as they are populated by the pump energies as Supplementary Fig. 8 shows. S2 and S3 states are instantly populated, and they depopulate completely within the first 100fs.

At the same time, while S1 state is also instantly populated by the pump, it populates even more within the first 100fs. We believe that this later population of S1 state originated from S2 and S3 states' depopulation as the time constants seems to agree.

Supplementary References

1. Miranda, M. *et al.* Characterization of broadband few-cycle laser pulses with the d-scan technique. *Opt. Express* **20**, 18732–18743 (2012).
2. Sytcevich, I. *et al.* Characterizing ultrashort laser pulses with second harmonic dispersion scans. *J. Opt. Soc. Am. B* **38**, 1546–1555 (2021).
3. Nelson, T., Fernandez-Alberti, S., Roitberg, A. E. & Tretiak, S. Nonadiabatic excited-state molecular dynamics: Modeling photophysics in organic conjugated materials. *Acc. Chem. Res.* **47**, 1155–1164 (2014).
4. Nelson, T., Fernandez-Aberti, S., Chernyak, V., Roitberg, A. E. & Tretiak, S. Nonadiabatic excited-state molecular dynamics modeling of photoinduced dynamics in conjugated molecules. *J. Phys. Chem. B* **115**, 5402–5414 (2011).
5. Clark, J., Nelson, T., Tretiak, S., Cirmi, G. & Lanzani, G. Femtosecond torsional relaxation. *Nat. Phys.* **8**, 225–231 (2012).
